# Human papilloma virus and breast cancer: the role of inflammation and viral expressed proteins

**DOI:** 10.1186/s12885-019-5286-0

**Published:** 2019-01-14

**Authors:** Niloofar Khodabandehlou, Shayan Mostafaei, Ashkan Etemadi, Amir Ghasemi, Mehrdad Payandeh, Shima Hadifar, Amir Hossein Norooznezhad, Anoshirvan Kazemnejad, Mohsen Moghoofei

**Affiliations:** 1 Department of Internal Medicine, Faculty of Medicine, Iran University of Medical Sciences, Tehran, Iran; 2 Department of Community Medicine, Faculty of Medicine, Alborz University of Medical Sciences, Tehran, Iran; 3 Department of Biostatistics, Faculty of Medical Sciences, Tarbiat Modares University, Tehran, Iran; 4 Department of Biology, Faculty of Science, Shahrekord University, Shahrekord, Iran; 5 Department of Materials Science and Engineering, Sharif University of Technology, Tehran, Iran; 6 Cancer Research Center, Kermanshah University of Medical Sciences, Kermanshah, Iran; 7 Department of Mycobacteriology & Pulmonary Research, Pasteur Institute of Iran, Tehran, Iran; 8 Regenerative Medicine Research Center, Kermanshah University of Medical Sciences, Kermanshah, Iran; 9 Department of Microbiology, Faculty of Medicine, Kermanshah University of Medical Sciences, Sorkheh-Ligeh Blvd, P. O. Box: 6716777816, Kermanshah, Iran

**Keywords:** Breast cancer, Human papilloma virus, Risk factor, Tumor development, Inflammation

## Abstract

**Background:**

Breast cancer is currently the most common neoplasm diagnosed in women globally. There is a growing body of evidence to suggest that human papillomavirus (HPV) infection may play a key role in invasiveness of breast cancer. The aim of this study was to determine the presence of HPV in patients with breast cancer and its possible association with cancer progression.

**Methods:**

Breast specimens were collected from 72 patients with breast cancer and 31 healthy controls. The presence of HPV was investigated by polymerase chain reaction (PCR) and genotyping was performed for positive cases. We also evaluated the viral factors such as E6, E2, and E7 in HPV positive cases. Enzyme-linked immunosorbent assay (ELISA (and Real-time PCR techniques were used to measure the expression level of anti-carcinogenic genes, such as *p53*, retinoblastoma (*RB*), breast and ovarian cancer susceptibility gene (*BRCA1*, *BRCA2)* and inflammatory cytokines, including tumor necrosis factor α (TNF-α), transforming growth factor β (TGF-β), nuclear factor-kB (NF-kB), and different interleukins [ILs] (IL-1,IL6, and IL-17).

**Results:**

The HPV DNA was detected in 48.6% of breast cancer samples, whereas only 16.1% of controls were positive for HPV. We observed statistically significant differences between breast cancer patients and HPV presence (*P = 0.003*). HPV type 18 was the most prevalent virus genotype in patients. The expression of *P53*, *RB*, *BRCA1*, and *BRCA2* were decreased in patients with HPV-positive breast cancer as compared to HPV-negative breast cancer and healthy controls. (All *P-values* were less than 0.05). The presence of the HPV was associated with increased inflammatory cytokines (IL-1, IL-6, IL-17, TGF-β, TNF-α, and NF-kB) and tumor progression.

**Conclusion:**

The present study demonstrated that HPV infection may implicate in the development of some types of breast cancer.

## Background

Breast cancer is one of the most common cause of cancer deaths among women in both developing and developed countries. Moreover, mortality of this cancer is much more than both colorectal and lung cancers [1–3]. In the past two decades, the breast cancer rate has been increased worldwide with a considerable pace which has been suggested to be due to increasing known and/or unknown risk factors (RFs) of this cancer. A group of these RFs could be infectious agents which play a key role as carcinogens or promoters [4–6]. Recent studies have identified that some viruses, especially human papilloma virus (HPV), are among the RFs for the development of breast cancer, suggesting a strong association between HPV and breast cancer [7–10]. Many researches have been done on association between human papilloma virus (HPV) and cervical cancer and this issue is well accepted that HPV has the strong causal link with this cancer [9, 10]. For the first time in 1992, the association between breast cancer and HPV was reported by *Lonardo* et al. [11]. The HPV is a non-enveloped DNA virus which belongs to the *Papillomaviridae* family with over 150 types [12]. It has been shown that at least a few types of HPV such as 6, 11, 15, 16, 18, and 33 are related to breast cancer [13, 14]. The genome of such viruses are divided into three main segments; long control region (LCR), early region (E) which encoding *E1, E2, E4–E7*, and late region (L) consisting of *L1* and *L2* [15]. E6 and E7 proteins, the oncoproteins, mainly act as stimulators of host cell proliferation [16]. E6 protein is a greatly important functional protein which interacts with p53 and BCL2 antagonist/killer (BAK 34) to increase the chromosomal instability and cellular resistance to apoptosis [17]. E7 protein interacts with retinoblastoma (RB) resulting in E2F release, a transcription factor which promotes cell proliferation. E7 up-regulates S-phase genes, cyclin A, and cyclin E but,contrarily, inhibits the cyclin-dependent kinase inhibitors such as the cyclin-dependent kinase inhibitor (WAF 1), known as p21, and Kinesin-like protein (KIP 1), known as p27 [16, 18]. Other equally important cellular factors, which interact with HPV proteins, are breast and ovarian cancer susceptibility gene-1 (*BRCA1*) and *BRCA2*. These genes are known for their tumor suppressor products, which prevent tumor development by repairing DNA damages. These proteins activate c-Jun N-terminal kinase/stress-activated protein kinase (JNK/ SAPK) that eventually lead to apoptosis [19–21]. E7 and E6 interact with BRCA1 and antagonize several functions of BRCA1 [22].

Other factors such as inflammation have been shown to be involved in breast cancer progression. Chronic inflammation, which can be caused by persistent virus infections, is mediated by different cytokines and reactive oxygen nitrogen species (RONS). This chronic situation could suppress the antitumor immunity, promote metastasis development, and contribute to tumor progression [23–25]. Moreover, inflammation could enhance tumor progression which results in tissue remodeling, induction of the growth factors and angiogenesis [6]. Different cytokines such as transforming growth factors like beta (TGF-β), interleukin 1 (IL-1), IL-6, and IL-17 could stimulate breast cancer cell proliferation and/or invasion [26]. IL-6 is one of the inflammatory cytokines involved in tumor growth by evoking anti-apoptotic response and stimulating tumor development [27]. TGF-β is a multifunctional cytokine involved in regulating many processes including differentiation, proliferation, and apoptosis of cancer cells. This cytokine seems to be the most extensively studied factor in breast cancer molecular studies [24]. During the inflammation, increased levels of IL-1 could induce breast cancer progression via angiogenesis, cell proliferation, and inhibition of apoptosis [28, 29]. Tumor necrosis factor α (TNF-α), is another inflammatory cytokine expressed in high amounts and involved in breast cancer [24]. TNF-α, IL-6 and TGF-β promote the production of IL-17 which affects chronic inflammatory responses and thus tumor development [25]. NF-κB (nuclear factor kappa-light-chain-enhancer of activated B cells) is a protein which plays a critical role in regulating the immune response against infections. This feature seems to be the main linker between tumor development and inflammation [30]. Several studies have shown that NF-κB has a direct association with tumor initiation and cancer progression [31]. High levels of RONS in cancer tissue can significantly promote tumor development and metastasis [32, 33]. Chronic infection and inflammation are the cause of 20–25% of all human cancers [34]. Therefore, investigating the role of infection and inflammation in tumor initiation and development has been attracted intensive scientific interests in the fields of oncology and virology.

In the present case-control study, we aimed to determine the presence of HPV in breast cancer tissues and to evaluate the possible association between HPV infection and breast cancer development.

## Methods

### Samples, methodology, and ethical standards

This multi-central case control study was performed between January 2015 and March 2016 in the Kashani Hospital (Shahrekord, Iran) and Rasul-e Akram Hospital (Tehran, Iran). According to the inclusion and exclusion criteria, 72 breast specimens were collected, and all tissue samples were immediately snap frozen in liquid nitrogen and stored at − 80 °C. Inclusion criteria were defined as; women with approved histopathological (biopsy) evidence(s) of breast cancer, accessibility of fresh samples, native patients of Shahrekord and Tehran cities. Also, different parameters such as past or current medical history of chemotherapy and/or radiotherapy, being pregnant, biologic anti-cancer therapies, and systemic inflammatory disease such as rheumatoid arthritis were defined as exclusion criteria. No limitations in age, type of breast cancer, and tumor size or stage were considered for the patients. In addition, 31 normal breast tissue samples, obtained from breast reduction surgeries with normal histopathology results, were also examined as healthy controls (from both hospitals). All the controls were healthy women with no history of estrogen therapies, oral contraceptive consumption, cervical cancer, and smoking. For all the cases a carful breast examination was performed by an experienced surgeon. All the histopathology results were re-examined by two well experienced pathologists (double checking by M. Mogani and M. Khosravi) to certainly confirm the diagnosis. The stage of cancer, based on TNM system, was provided by consulting an expert cancer team consisting of an oncologist, a radiologist and a cancer surgeon. Tumor samples were classified histologically based on the World Health Organization (WHO) criteria [35]. Five paraffin-embedded pathologically proved cervical cancer samples were used as positive controls. All the participants signed a copy of consent form freely after verbal explanation of the aims and methods of this study according to their level of knowledge.

### HPV detection, genotyping and physical status

The DNA extraction was performed using “QIAamp Tissue Kit” according to the manufacturer’s instructions (QIAGEN, Hilden, Germany). A polymerase chain reaction (PCR)-based detection assay was employed to identify HPV, using primers for *L1* and *E7* genes [1]. Genotypes of HPV positive samples were determined by INNO-LiPA HPV Genotyping v2 test (Innogenetics, Ghent, Belgium) in strict accordance with the manufacturer’s instructions. For this test, distilled water and paraffin sections without tissue were used as negative controls for PCR and DNA extraction, respectively. Moreover, isolated genotypes (6, 11, 15, 16, 18, and 33) of cervical cancer samples, in CIN3 and cervical cancer model, were used as positive controls for amplification. The serial dilutions of the full-length HPV genome was prepared to provide the standard control for copy number of *E2* and *E6* genes [36].

### Expression level of cellular and viral factors

#### E6

Total RNA was extracted and purified from the tissue by using RNEasy Mini kit (QIAGEN, Hilden, Germany). Real-time PCR (RT-PCR) reactions were conducted with one step RT-PCR® kits (QIAGEN, Hilden, Germany) according to the manufacturer’s instructions. The used primers for amplifying the gene sequence for *E6* were [37]:

Forward 5′-GCAATGTTTCAGGACCCACA-3′

Reverse 5′-ACAGCATATGGATTCCCATCTC-3′.

#### p53

The level of p53 was assessedusing enzyme-linked immunosorbent assay (ELISA) using Abcam’s p53 Simple Step ELISA® Kit (Abcam, Cambridge, MA, USA) according to the manufacturer’s instructions.

#### E7

For cDNA synthesis, 1 microgram of extracted total RNA was reverse transcribed using the QuantiNova Reverse Transcription® Kit (QIAGEN, Hilden, Germany). The used primers and probe in *E7* gene amplification were [38]:

Forward primer: 5’-AAGTGTGACTCTACGCTTCGGTT-3’

Reverse primer: 5’-GCCCATTAACAGGTCTTCCAAA-3’

Probe: FAM-TGCGTACAAAGCACACACGTAGACATTCGTA-BHQ

#### RB

The expression level of RB gene was determined by Human Retinoblastoma ELISA® kit (Sigma-Aldrich, Saint Louis, USA) according to the manufacture’s protocol.

#### E2

Quantitative SYBR green TaqMan Universal PCR Master Mix® (QIAGEN, Germany) was used to monitor expression levels of *E2* genes. The used primers in *E2* gene amplification were [39]:

Forward primer: 5’-CTACGAATTCATGGAGACTCTTTGCCAACG-3′

Reverse primer: 5’-GATAGAATTCTCATATAGACATAAATCCAG-3′

#### BRCA1 and BRCA2

The expression level of BRCA1 and BRCA2 were measured by BRCA1 and BRCA2 ELISA Kits (Human) (MyBioSource, Inc. CA, USA) according to the manufacture’s protocol.

#### Cytokines and NF-kB evaluation

The levels of IL-1, IL-6, IL-17 and NF-kB were measured using Human IL-6 ELISA® Kit, Human IL-1 beta ELISA® Kit, Human IL-17 ELISA® Kit, and NFkB p65 Transcription Factor Assay® Kit **(**Abcam, Cambridge, MA, USA), respectively, according to the manufacturer’s instructions. Moreover, the amount of TGF-β and TNF-α were measured by Human TGF-beta 1 Quantikine ELISA® Kit (Minneapolis, MN, USA) and Human TNF Alpha PicoKine™ ELISA Kit (Boster Biological Technology, Pleasanton CA, USA), respectively, according to the manufacturer’s instructions.

#### Reactive oxygen species and reactive nitrogen species

The RONS level was assessed by OxiSelect™ Intracellular ROS/RNS Assay kit (Cell Biolabs, Inc., San Diego, CA), following the protocol.

#### Statistical methods

Normality test was performed using Kolmogorov–Smirnov test for continuous variables. The two-independent samples t-test (Mann-Whitney non-parametric test) was conducted to compare the central tendency (e.g. mean for normal and median for non-normal gene expression) of gene expressions in such two groups. Correlation analysis was also carried outby Eta-squared coefficient. To identify the linear dependencies between two sets of the variables, the Canonical Correlation Analysis (CCA) was applied. Generalized linear model (logistic regression) was used to recognize the association between the presence of HPV and breast cancer. Moreover, for this test odds ratio (OR) as the effect size with 95% confidence intervals (95% CI) was measured. False discovery rate was corrected by Benjamini-Hochberg approach for multiple comparisons. All data were finally analyzed using IBM SPSS version 21.0 (SPSS, Chicago, IL, USA) and GraphPad Prism version 6 (La Jolla, CA, USA). Any *P-value* less than 0.05 were considered statistically significant.

## Results

In this study, 72 female breast cancer cases, including 9 (12.5%), 20 (27.8%), 32 (44.4%), 3 (4.2%), and 8 (11.1%) patients with medullary carcinoma, invasive lobular carcinoma, invasive and in-situ ductal carcinoma, mucinous carcinoma, and tubular carcinoma were examined. The average age of the patients was 48.86 ± 10.95 years, ranging from 30 to 81 and for the controls it was 48.97 ± 9.22 years old (ranged from 35 to 72), which was similar to the age of the patients (*P* = 0.76) (Table 1).

**Table 1:** Comparison of participants’ characteristics between cases and controls

Characteristics		Breast cancer group (*N* = 72)	Healthy controls (*N* = 36)	*P*-value	OR (95% CI)
Age (Year)		48.86 ± 10.95	48.97 ± 9.22	0.76	–
HPV	Presence	35 (48.6%)	5 (16.1%)	0.003	4.92 (1.70–14.24)
	Absence	37 (51.4%)	26 (83.9%)	–	
Stage of Cancer	Ia, Ib	2 (2.7%), 9 (12.5%)	–	–	–
	IIa, IIb	20 (27.8%), 19 (26.3%)	–	–	–
	IIIa, IIIb,	5 (6.9%), 9 (12.5%),	–	–	–
	IIIc	6 (8.3%)			
	IV	2 (2.7%)	–	–	–
Type of Cancer	Ductal	32 (44.4%)	–	–	–
	Lobular	20 (27.8%)	–	–	–
	Medullary	9 (12.5%)	–	–	–
	Tubular	8 (11.1%)	–	–	–
	Mucinous	3 (4.2%)	–	–	–
Genotype	Negative (−)	35 (48.6%)	26 (83.9%)	0.037	–
	HPV-18	16 (22.2%)	3 (9.7%)		
	HPV-16	13 (18.1%)	2 (6.5%)		
	HPV-33	4 (5.6%)	–		
	HPV-11	3 (4.2%)	–		
	HPV-6	1 (1.4%)	–		

HPV DNA was detected in 35 out of 72 patients (48.6%) and 5 out of 31 healthy controls (16.1%). The presence of HPV infection in the breast cancer control was found to be statistically significant (*P* = 0.003). The odds of breast cancer incidence in the HPV positive group was 4.92 (95% C. I: 1.699–14.238), which was significantly more than HPV negative group. In the breast cancer group, five different genotypes were detected, namely, 18 (*N* = 16, 22.2%), 16 (*N* = 13, 18.1%), 33 (*N* = 4, 5.6%), 6 (N = 1, 1.4%), and 11 (*N* = 3, 4.2%), while, in the healthy control group only two genotypes was observed; 18 (N = 3, 9.7%) and 16 (*N* = 2, 6.5%) (Table 1). The papillomavirus *E2* gene expression was absence in 30 cases of breast cancer group (86%, *P* = 0.006), therefore the genome of HPV *E2* negative group was in an integrated form. However, the *E2*/*E6* ratio was lower than 1 which indicates that HPV was in both episomal and integrated forms known as mixed form (*N* = 5, 14%). Also, the *E2*/*E6* ratio in positive control group (5 cervical cancer samples), showed that only 20% (ration 1) was integrated and the other were episomal form, 80% (ratio 4) (*E2*/*E6* ≥ 1(. The *E2*/*E6* ratios and viral physical status in different cancer types and stages are compiled in Table 2. HPV DNA was detected in 16 patients (45.7%) with ductal carcinoma (highest ratio), and no patients (0%) with mucinous carcinoma (lowest ratio) was observed. Moreover, no significant association between histological types of breast cancer and HPV infection was detected (*P* = 0.32). A significant difference was found in the incidence of HPV infection between cases and controls (*P* = 0.003). Also, the association of HPV genotypes and occurrence of breast cancer was statistically significant (*P* = 0.037). The frequency distribution of breast cancer stages and genotypes between HPV positives and HPV negatives controls were statistically different and the association of HPV with stages of breast cancer and genotypes was also significant (*P* = 0.045 and *P* < 0.001, respectively) (Table 3).

**Table 2:** Physical status of HPV genome in cases and controls

	Cases (%)		Controls (%)	Total number	*P*-value
Integrated	30/35 (86%)		1/5 (20%)	31/40 (77.5%)	0.006
	Stages:	Types:			
		Ductal (*n* = 11)			
	I (n = 3)	Lobular (n = 4)			
	II (n = 3)	Medullary(*n* = 6)			
	III (*n* = 9)	Tubular (n = 5)			
	IV (*n* = 15)	Mucinous (n = 4)			
Episomal	–		4/5 (80%)	4/40 (10%)	NA
Mixed	5/35 (14%)		–	5/40 (12.5%)	NA
	Stages:	Types:			
	I (n = 3)	Ductal (n = 3)			
	II (n = 2)	Lobular (*n* = 0)			
	III (n = 0)	Medullary(n = 1)			
	IV (n = 0)	Tubular (n = 1)			
		Mucinous (n = 0)			

NA: Not applicable

**Table 3:** Comparison of participants’ characteristics between HPV positive and HPV negative groups in breast cancer cases

Characteristics		HPV+	HPV-	P-value	OR (95% CI)
Age (Year)		48.00 ± 11.11	49.46 ± 9.99	0.49	–
Stage of Cancer	I	4 (11.5%)	7 (18.9%)	Ref.	Ref.
	II	15 (42.8%)	24 (64.9%)	0.89	1.09 (0.27–4.38)
	III	14 (40%)	6 (16.2%)	0.076	4.08 (0.86–19.37)
	IV	2 (5.7%)	0	0.22	NA
Type of Cancer	Tubular	5 (14.3%)	3 (8.1%)	Ref.	Ref.
	Ductal	16 (45.7%)	16 (43.2%)	0.53	0.60 (0.12–2.94)
	Medullary	4 (11.4%)	5 (13.5%)	0.46	0.48 (0.06–3.35)
	Lobular	10 (28.6%)	10 (27%)	0.55	0.60 (0.11–3.21)
	Mucinous	0	3 (8.1%)	0.15	NA
Genotype	Negative (−)	0	61 (96.8%)	< 0.001	NA
	HPV-18	18 (45%)	1 (1.6%)	Ref.	Ref.
	HPV-16	15 (37.5%)	0	0.58	NA
	HPV-33	4 (10%)	0	0.85	NA
	HPV-11	2 (5%)	1 (1.6%)	0.17	0.11 (0.005–2.55)
	HPV-6	1 (2.5%)	0	0.44	NA

Ref: reference level for the categorical variable, NA: not applicable

**Fig. 1:**
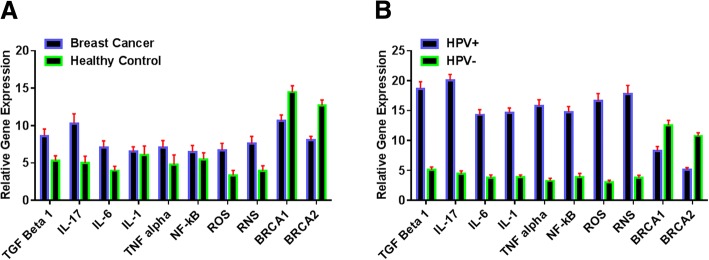
Comparison of inflammation factors, BRCA1 and BRCA2 expression level: (**a**) in breast cancer with control, (**b**) in HPV positive with HPV negative samples

The expression levels of p53 and RB was found to be reduced in HPV-positive breast cancer group compared to HPV-negative breast cancer and normal healthy controls (*P* < 0.001, *P* < 0.001, *P* = 0.033, respectively) (Table 4). In addition, there was a direct association between the decreased expression of the tumor suppressor genes (p53, RB, *BRCA1* and *BRCA2)* and the progression stage of breast cancer. The results showed a significant negative association between expression of *E6* and p53 (*P = − 0.743, P* < 0.001), and similar association between the expression level of *E7* and RB (*P = − 0.805*, *P* < 0.001) was detected. According to CCA result, standardized canonical correlation coefficient between the expressions of inflammatory factors and viral proteins (*E2*, *E6* and *E7*) were statistically significant (*P = 0.692* and *P* = 0.009). The expression levels of different inflammatory factors including IL-1, IL-6, IL-17, TGF-β, TNF-α, NF-κB, and RONS were statistically higher in HPV- positive breast cancer patients than control samples and HPV-negative breast cancer patients. More details are presented in the Tables 5, 6 and Fig. 1.

**Table 4:** Comparison of *RB* and *p53* expression levels between patients with breast cancer/ HPV positive groups and control samples/ HPV negative groups

Expression level	Breast Cancer (*N* = 72)	Control (*N* = 31)	Fold Change	Adjusted *P*-value	HPV positive (*N* = 40)	HPV negative (*N* = 63)	Fold Change	Adjusted *P*-value
*RB*	7.89 ± 5.74	10.65 ± 6.39	0.74	0.033	3.25 ± 3.29	12.19 ± 4.67	0.27	< 0.001
*P53*	9.81 ± 7.51	17.19 ± 6.02	0.57	0.001	5.30 ± 4.46	16.30 ± 6.41	0.33	< 0.001

**Table 5:** Comparison expressions level of *TGF-β, IL-17, IL-6, IL-1, TNF-α, NF-κB, ROS, RNS, BRCA1* and *BRCA2* in breast cancer, control samples, positive HPV, and negative HPV samples

Expression level	Breast Cancer (*N* = 72)	Control (*N* = 31)	Fold Change	Adjusted *P*-value^*^	HPV positive(*N* = 40)	HPV negative(*N* = 63)	Fold Change	Adjusted *P*-value^*^
TGF-β	12.40 ± 9.11	5.29 ± 3.68	2.34	0.0013	18.65 ± 7.53	4.94 ± 3.06	3.75	0.001
IL-17	12.61 ± 9.46	4.97 ± 4.29	2.54	0.0013	20.05 ± 6.28	4.13 ± 3.33	4.85	0.001
IL-6	9.32 ± 7.27	3.97 ± 3.29	2.35	0.0013	14.30 ± 5.58	3.52 ± 3.22	4.06	0.001
IL-1	8.90 ± 6.21	6.10 ± 4.48	1.46	0.0103	14.65 ± 5.06	3.87 ± 2.30	3.78	0.001
TNF-α	9.40 ± 7.56	4.77 ± 3.28	1.97	0.0013	15.83 ± 6.33	3.05 ± 3.01	5.19	0.001
NF-κB	9.26 ± 7.67	5.48 ± 4.91	1.69	0.0290	14.75 ± 5.97	3.92 ± 3.51	3.76	0.001
ROS	10.08 ± 8.87	3.35 ± 3.06	3.01	0.0013	16.38 ± 7.29	2.78 ± 2.47	5.89	< 0.001
RNS	11.32 ± 9.84	3.94 ± 2.88	2.87	0.0013	17.82 ± 8.82	3.56 ± 2.83	5.01	< 0.001
BRCA1	9.58 ± 5.35	14.45 ± 4.58	0.66	0.0013	8.32 ± 4.43	12.77 ± 5.58	0.65	< 0.001
BRCA2	7.23 ± 3.84	12.66 ± 4.03	0.57	0.0013	5.15 ± 1.92	11.22 ± 3.79	0.46	< 0.001

* FDR correction for multiple comparisons by Benjamini-Hochberg method

**Table 6:** Associations between expression level of *TGF-β, IL-17, IL-6, IL-1, TNF-α, NF-κB, ROS, RNS, BRCA1* and *BRCA2* with breast cancer and presence of HPV

Expression level	Breast Cancer		Presence of HPV	
	Correlation Coefficient	Adjusted *P*-value^*^	Correlation Coefficient	Adjusted *P*-value^*^
TGF-β	0.608	0.0016	0.877	< 0.001
IL-17	0.618	0.0087	0.953	< 0.001
IL-6	0.464	0.0016	0.959	< 0.001
IL-1	0.497	0.0410	0.949	< 0.001
TNF-α	0.602	0.0071	0.971	< 0.001
NF-κB	0.418	0.0155	0.953	< 0.001
ROS	0.613	< 0.001	0.958	< 0.001
RNS	0.633	< 0.001	0.956	< 0.001
BRCA1	0.831	< 0.001	0.797	< 0.001
BRCA2	0.736	< 0.001	0.952	< 0.001

* FDR correction for multiple comparisons by Benjamini-Hochberg method. Eta-squared considered as an effect size of correlation

## Discussion

Breast cancer incidence and mortality rates are increasing markedly worldwide. This highlights the importance of identifying new RFs that are related to breast cancer development, in order to prevent or treat the disease [40]. Several factors are involved in initiation and development of cancer, among which are critically important biological carcinogens such as viral infections [41]. Viral infections have been shown to be involved in approximately 18–20% of cancers [40]. For example, HPV can induce inflammation due to some of its features including production of oncogenic proteins. This makes HPV a strong suspect for initiation and development of breast cancer [42].

In this study, among 72 evaluated samples, HPV DNA was found in 48.6% (*n* = 35) of breast cancer samples. Several investigations have reported detecting HPV in breast cancer patients and its prevalence with a vast range from 4 to 86% [43]. A recent evaluation conducted by *Sigaroodi* et al. (2012, Iran) has shown a high frequency of HPV DNA in breast cancer patients (25.9%) in contrast to the women with non-cancerous condition (2.4%). According to their results, the HPV genotypes 16 and 18 with the accumulated prevalence of 53.34% in breast cancer patients were the most predominant. This study also showed that breast cancer in Iranian women was associated with HPV infection (OR 14.247, 95% CI 1.558–130.284; *P* = 0.019) [14]. Another investigation which performed by *Salman* et al. (2017, UK) reported the presence of HPV genome (42%) in breast cancer patients, concluding that high expression level of *E6* and *E7* and their interactions with cellular factors can led to breast tumor development [40]. These results are consistent with our results and also with earlier studies on cervical cancer [44, 45]. Other investigations have reported different prevalence of HPV in breast cancer patients as follows; 41.6% (2015, Venezuela), 40% (2013, Mexico) and 21% (2008, Japan) [36, 46, 47]. Our result and other studies have been compared in Table 7. It is noteworthy that despite using viral DNA detection method to show the presence of the virus, this does not demonstrate the active infection. [40]. Several studies have shown the expression of the HPV proteins in breast tissue is a reliable marker to detect the active infection. For instance, it has been demonstrated that the expression level of *E7* in breast cancer tissue was higher than healthy controls and patients with early stages of cancer [40]. Therefore, we assessed the expression level of *E6* and *E7* to evaluate the viral activity.

**Table 7:** Evaluation of differences in prevalence of HPV infected breast cancer patients among different studies

Study	Year/Country	HPV^+^ patients	HPV^+^ controls	Most prevalent HPV
Current study	2017/Iran	48.6%	16.1%	HPV-18 (22.2%)
Islam et al.	2017/India	63.9%	9.5%	HPV-16 (69%)
Salman et al.	2017/UK	42%	17%	HPV-39 (20%)
Fernandes et al.	2015/ Venezuela	41.6%	NA	HPV-51 (30.7%)
Herrera-Goepfert et al.	2013/Mexico	40%	NA	HPV-16 (87.5%)
Sigaroodi et al.	2012/Iran	25.9%	2.4%	HPV-16 and 18 (both 25%)
Khan et al.	2008/Japan	21%	NA	HPV-16 (92%)
Yu et al.	2000/China	43.8%	NA	HPV-33 (43.8%)

HPV 16 and 18 genotypes are considered as two of the most common virus genotypes that can be found in cancers worldwide [36, 47]. Other types of HPV including 6, 11, 33, 35, 39, 45, 51 and 59 have been also detected in breast cancers patients [40, 46, 48]. For the first time, *Yingyan Yu* et al. (2000, China) reported the detecting HPV-33 in 43.8% of their evaluated patients with breast cancer. However, they did not report the presence of HPV-16 and HPV-18 DNA in their cases [48]. In the current study, we reported the prevalence of HPV-33 to be 5.6% in breast cancer samples but not in any of healthy controls. There could be several hypotheses addressing this difference, prevalence and genotypes distribution of HPV, including geographical, sample size, sampling, and methodological differences [36, 49]. Among all the mentioned reasons, it seems that the main reason could be the difference in the geographical areas which may cause variety in the prevalence of HPV type distribution. Another important factor is the sensitivity of methods used to detect viral genome. For instance, the use of different primer sets may result in highest sensitivity and specificity. In the current study, two sets of primers were used for *L1* and *E6/E7*, since *L1* gene is frequently lost during HPV integration into the host genome [50]. Although different methods such as in situ hybridization (ISH) could be used for HPV DNA detection, but this method may not detect HPV DNA in some samples. Therefore, it has been suggested that the PCR can be more sensitive than ISH [49]. A study has been conducted to compare the sensitivity of PCR and ISH methods for the detection of HPV in patients with breast cancer. This investigation was considered since they used PCR method to show the presence of HPV DNA in 46% of breast cancer cases, while using ISH resulted in only one positive case [51]. Our results showed that in the group of HPV DNA positive patients, the ductal carcinoma was more frequent than other types of cancer (*N* = 16, 45.7%). It was also accompanied with the lower incidence of mucinous carcinoma (0%). Also, we showed that the highest HPV incidence was similar to the recent study investigating on the role of HPV in breast carcinogenesis in the UK. In contrast, the prevalence of HPVs DNA in lobular carcinoma was found to be the lowest [40].

The *E2*/*E6* ratio was used to determine the physical status of HPV genome. When the ratio is equal to zero, larger than zero and smaller, equal to or higher than 1, HPV genome is integrated, made up from both episomal and integrated forms and episomal form, respectively [36, 52]. Previous studies have shown that physical status of HPV DNA in cervical cancer is considered as a marker of tumor development [52]. *Khan* et al. detected HPV DNA in 21% of evaluated breast cancer samples. They demonstrated that all the HPV genome was considered integrated into the host genome except for one case which was a mixed form [36]. *Islam and colleagues* reported a prevalence of 63.9% for HPV in patients with breast cancer, demonestirating that 87.5% of their patients with positive HPV had integrated genome and 4.2% were in episomal form [53]. In the present study, according to the *E2*/*E6* ratio, 30 (86%, *P* = 0.006) and 5 (14%) of HPV genomes were integrated and mixed form, respectively. Our results demonstrated that *E2/E6* ratio was significantly different in the tumor types and stages. Most integration and mixed form were detected in ductal form of tumor and stages III and IV. Such high rate of integration is accompanying with tumor development because this finally leads to increasing the expression level of *E6* and *E7*. This issue has already been proven in Cervical Cancer [40]. One of the limitations in our study was to use *E2*/*E6* ratio for determination of physical status of HPV genome while *Zhang* et al. have demonstrated that *E2/E6* or *E2/E7* is less sensitive and predictive than *E2/E6E7* for determination of physical status of HPV genome in cervical cancer [54]. Therefore, in our study, the percentage of integrated HPV genomes was probably more than the percentage we reported. On the other hand, the multiple E1-L1/E6E7 ratio analysis needs four more PCR reactions than E2/E6E7 ratio analysis. Further studies is required to estimate the cost-effectiveness of this modification, which can be considered as a drawback. [54]. To the best of our knowledge, this is the first study reporting the physical status of HPV genome in breast cancer tissue in Middle East.

There are numerous distinct stages from the beginning of viral infection to the tumor development. For example, the progression of cervical tumors in patients infected by HPV are including these models: (1) HPV infection CIN 1, 2; (2) persistent HPV-infection, CIN 1, 2; (3) CIN 3; (4) cervical cancer. These stages are affected by different cofactors [9, 55]. The role of HPV in initiation and development of breast cancer can be discussed from two aspects: (1) Direct role; interactions of viral proteins with key regulator proteins of the cell, (2) Indirect role; induction of inflammation.

Through targeting p53 and RB, oncoproteins of HPV such as E6 and E7 could disrupt the cell cycle, initiate malignant transformation and finally lead to tumor development [44]. This is inevitable since p53 and RB play crucial roles in controlling cell cycle and genome repair, and therefore interference and degradation of these proteins may lead to uncontrolled cellular proliferation and finally cancer. Herein, it was demonstrated that compared to controls, the expression levels of p53 and RB in cancer samples were decreased significantly (*P = 0.001* and *P = 0.033* respectively*)*. Also, the expression levels of p53 and RB were compared between HPV positive and HPV negative patients diagnosed with breast cancer and it was showed that these levels were significantly reduced in HPV-positives breast cancer patients compared to HPV-negatives breast cancer patients (both *P < 0.001*).

In the breast, as well as all other tissues, the BRCA1 and BRCA2 are expressed and involved in repair process of damaged DNA and any reduction or disruption of these two proteins could lead to cancer [56]. It has been shown that E6 and E7 proteins are able to interact with the BRCA1 (as the antagonists) and alter its activity. Moreover, BRCA1 interacts with RB and p53. This interaction is required for the RB functioning in G_1_ checkpoint of cell cycle. Also, BRCA1 acts as a co-activator of p53-mediated transcription [22]. Thus, HPV proteins (E6 and E7), may influence RB and p53 functions with indirect interference through BRCA1 pathway. In addition, we demonstrated that the expression levels of BRCA1 and BRCA2 were reduced in breast cancer tissue in comparison to the healthy controls (for both *P = 0.0013*). Moreover, in HPV-positive breast cancer patients BRCA1 and BRCA2 were decreased (both *P < 0.001*) compared to the HPV-negative group., suggesting that the role of HPV in breast cancers could be accomplished through interacting with these proteins.

Previous studies indicated that onco-viruses can be considered as a cause some types of cancers, but these infections seem to be just a prerequisite and viral infections can only provide some of the conditions which is necessary for carcinogenesis. Although, high-risk HPV types can lead to transform the human keratinocytes into cancer cells in vitro, without any additional factors [57], other co-factors such as chronic inflammation, environmental mutagens, and immunosuppression are required to carcinogenesis [58, 59]. Several epidemiological and clinical investigations have demonstrated that certain pathogens leading to persistent infection(s) are strongly correlated with cancer prevalence [60]. Persistent viral infections usually cause chronic inflammation through different factors such as induction of RONS production and producing mitogenic and angiogenic factors [25, 61]. Inflammation could be considered as a double-edged sword; in the initial steps it is crucial for provoking anti-tumor responses by the immune system and after that, it favors tumor development by triggering angiogenesis [23]. Recent investigations indicated that the inflammation is the major hallmark for tumor progression [60, 62]. Among the inflammatory cytokines, IL-1, TGF-β, and IL-6 are responsible for cancer cells proliferation and invasion through activation of NF-κB. TNF-α, another inflammatory cytokine, is responsible in the main pathways of tumor inflammation. Moreover, it has been shown that TNF-α--NF-κB axis is related to the invasiveness and malignant behavior of breast cancer cells. TNF- α, IL-1, TGF-β, and IL-6 are able to induce the expression of many angiogenic growth factors in tumor such as vascular endothelial growth factor (VEGF) [24]. Also, IL-17 is related to the cancer cell survival and invasion as well as regulation of angiogenesis [63]. The present study clearly showed that the expression of inflammatory factors including IL-1, IL-6, IL-17, TNF-α, TGF-β, NF-κB and RONS in HPV-positive breast cancer patients were higher than HPV-negative breast cancer patients and healthy controls (Fig. 1 and Table 5). This indicated a significant role of inflammation in cancer induction among HPV infected patients with breast cancer. The increased inflammatory status caused by HPV may increase tumor development and tumor cells’ survival as well as angiogenesis [22], and consequently this lead to cancer cells proliferation and tumor metastasis [23]. Taken together, it seems that the detected inflammation in these patients may be related to viral infection, persistent infection, and its proteins. Despite all the results of this study, the role of HPV in breast cancers is still questionable. To the best of our knowledge, this is the first study reporting the association between inflammation and HPV in breast cancer patients.

## Conclusion

In the current study, HPV genome was detected in 48.6% of breast cancer samples among which most (82.8%) were at stage II and III. Being infected with HPV as a risk factor could directly or indirectly interfere with certain cellular mechanisms which lead to tumorigenesis and cancer development. We demonstrated that HPV is associated with breast cancer development, although the role of HPV in breast cancers is still questionable and further research is required to investigate, in more detail, the role of HPV infection in breast cancer.

## Abbreviations

BAK: BCL2 antagonist/killer
BRCA: Breast and ovarian cancer susceptibility gene
CI: Confidence interval
ELISA: Enzyme-linked immunosorbent assay
HPV: Human papilloma virus
IL: Interleukin
JNK/SAPK: c-Jun N-terminal kinase/stress-activated protein kinase
KIP: Kinesin-like protein
NF-κB: Nuclear factor κB
PCR: Polymerase chain reaction
RB: Retinoblastoma
RF: Risk factor
RONS: Reactive oxygen nitrogen species
RT-PCR: Real-time PCR
TGF-β: Transforming growth factor β
TNF-α: Tumor necrosis factor α
VEGF: Vascular endothelial growth factor
